# Ameloblastic Fibroodontoma: Uncommon Case Presentation in a 6-Year-Old Child with Review of the Literature

**DOI:** 10.1155/2017/9483738

**Published:** 2017-08-13

**Authors:** Anshad Mohamed Abdulla, G. Sivadas, L. K. Surej Kumar, C. S. Sheejith Hari Peeceeyen, Vaishnavi Vedam

**Affiliations:** ^1^Department of Paedodontics and Preventive Dentistry, International Medical University (IMU), Kuala Lumpur, Malaysia; ^2^Department of Paedodontics and Preventive Dentistry, Faculty of Dentistry, Asian Institute of Medicine, Science & Technology (AIMST) University, Kedah Darul Aman, Malaysia; ^3^Department of Oral and Maxillofacial Surgery, KIMS Hospital, Trivandrum, Kerala, India; ^4^Dental Services, KIMS Hospital, Trivandrum, Kerala, India; ^5^Department of Oral Pathology, Faculty of Dentistry, Asian Institute of Medicine, Science & Technology (AIMST) University, Kedah Darul Aman, Malaysia

## Abstract

Ameloblastic fibroodontoma is a benign mixed odontogenic neoplasm considered in patients with asymptomatic swelling and unerupted teeth that exhibit histologic features between ameloblastic fibroma and complex odontoma. Radiographically, this lesion appears as radiolucency admixed with focal radio opaque masses of irregular shapes and sizes. This lesion is confirmed by the presence of proliferating odontogenic epithelium, ectomesenchyme, and dental hard tissue formation on pathological analysis supplementing clinical and radiographic findings. As this tumour is less commonly seen in routine clinical practice, ameloblastic fibroodontoma with detailed orofacial features and periodic approach to its diagnosis is discussed. This paper reports a case of ameloblastic fibroodontoma of the mandible in a 6-year-old male patient with an uncommon case presentation and review of the literature.

## 1. Introduction

Odontogenic tumours are a diverse group of diseases ranging from simple hamartomas to neoplasms with metastatic potential [1]. Ameloblastic fibroodontoma (AFO) is a rare tumour characterized by proliferating odontogenic epithelium within primitive cellular ectomesenchymal tissue with/without hard tissue formation [2]. This tumour is characterized by well circumscribed, asymptomatic slow expansile swelling that is usually associated with unerupted or displaced tooth [3]. Initially it was termed as ameloblastic odontoma, but due to its exceptional mixed nature of growth and incidence of two types of odontogenic tumours with diverse histological and biologic behavior, World Health Organization (WHO) suggested this term to be inappropriate. Later, it was aptly named as ameloblastic fibroodontoma (AFO) by Hooker (1967) [4].

We present a case of ameloblastic fibroodontoma (AFO) in a 6-year-old male patient with distinctive orofacial manifestations, radiographic findings, histopathological report, and differential diagnosis with treatment to add to the existing knowledge.

## 2. Case Presentation

A 6-year-old male patient reported to the dental hospital with a chief complaint of swelling on the right side of face since 1 month. History revealed a slow growing and nontender swelling. Medical, personal, and family history was noncontributory. The patient was well oriented with stable vital signs. On extraoral examination, mild facial asymmetry due to swelling in the right body of mandibular region, with ill-defined margins, was evident. On palpation the swelling was asymptomatic and hard in consistency. Skin over the swelling was normal with no evidence of secondary changes. Restricted temporomandibular joint (TMJ) movements and palpable lymph nodes were evident.

**Figure 1:**
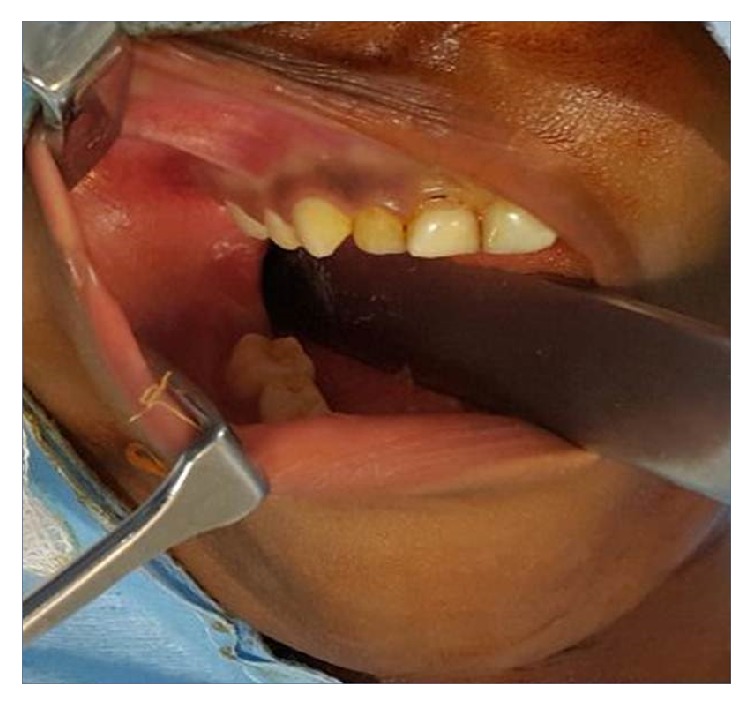
Intraoral examination reveals the obliteration of buccal sulcus adjacent to molar teeth with buccal and lingual cortical expansion.

**Figure 2:**
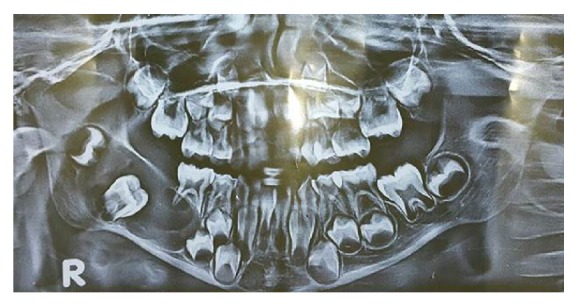
Orthopantomogram reveals radiopaque lesion surrounded by a radiolucent zone extending anteriorly from the lower first molar region to body of the mandible posteriorly on the right side.

On intraoral examination, obliteration of the buccal sulcus in molar region with buccal and lingual osseous cortical expansion was apparent (Figure 1). On palpation, the swelling was fluctuant, nontender, and fixed to the underlying bone. Orthopantomogram (OPG) exhibited a large radiopaque lesion surrounded by a radiolucent zone, which extended anteriorly from the lower first molar region to body of the mandible posteriorly on the right side (Figure 2). Routine blood investigations were also under normal limits which ruled off evidence of any systemic disorder.

**Figure 3:**
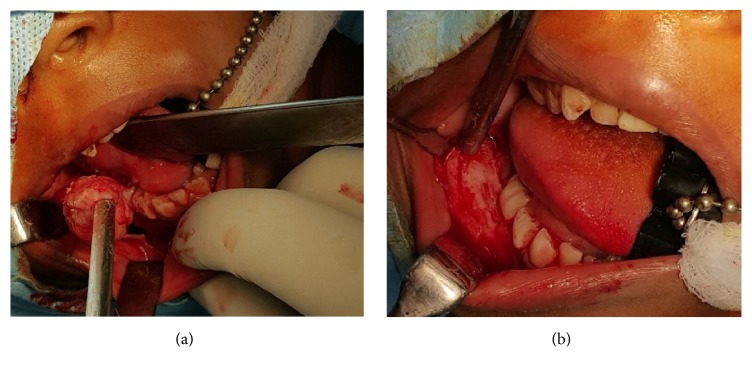
Surgical enucleation of the lesion under general anesthesia.

Based on clinicoradiographical findings, a provisional diagnosis of complex odontoma was made. The patient underwent enucleation of the lesion and curettage under general anesthesia. Tooth attached to the follicle around the lesion was removed. The cavity was irrigated carefully and the debris was detached. The flap was repositioned in the same location and suturing was done with vicryl 3.0 suture material (Figures 3(a) and 3(b)).

**Figure 4:**
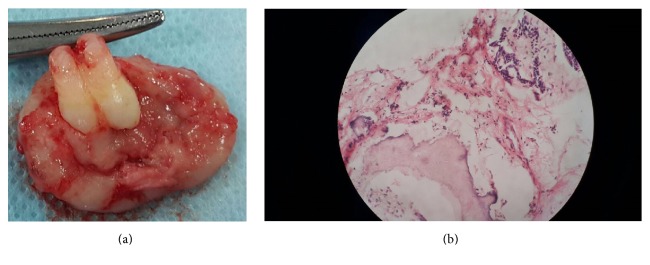
Histopathological specimen (a) reveals the presence of the lesional tissue in toto along with the attached tooth specimen. (b) Histopathology shows odontogenic epithelium with peripheral palisading nuclei resembling ameloblast-like cells and loosely arranged central cells, identical to stellate reticulum, embedded in a myxoid cell-rich stroma resembling the dental papilla. Focal areas of enamel and dentin are also present.

Surgical specimen was fixed in 10% formalin and subjected to pathological analysis. On gross examination the specimen consisted of a hard tissue mass with a soft tissue attachment. Routine histopathological examination revealed strands and islands of odontogenic epithelium with peripheral palisading nuclei resembling ameloblast-like cells and loosely arranged central cells, identical to stellate reticulum, embedded in a myxoid cell-rich stroma resembling the dental papilla. Dentin and enamel were also present (Figures 4(a) and 4(b)). Correlating the clinicoradiological and histopathological findings the lesion was diagnosed as ameloblastic fibroodontoma. Follow-up with this patient 2 years postoperatively exhibited good prognosis and no evidence of recurrences.

## 3. Discussion

Ameloblastic fibroodontoma has been conventionally classified as a benign mixed odontogenic tumour with limited cases documented in literature. This lesion is reported in approximately 0.3% to 1.7% of odontogenic tumours among different locations with 4.6% accounting to total number of paediatric cases. Children or teenagers with a mean age of 8–12 years are commonly affected with no obvious gender or anatomic site predilection. This lesion is frequently seen in the posterior border of the mandible (2.4 : 1) as in the present case [5].

Debate exists concerning the histogenesis of the ameloblastic fibroodontoma as a mixed odontogenic neoplasm. Previously, concept stated this lesion to be continuum of differentiation from ameloblastic fibroma to complex odontoma. However, the recent literature stated that ameloblastic fibroodontoma as a discrete entity but it can be histologically indistinguishable from immature complex odontoma due to the presence of abundant dental hard tissue formation [6]. According to the revised World Health Organization (WHO) classification, ameloblastic fibroodontoma is now considered as a benign tumour without invasive growth [7]. Philipsen et al. suggested the reduced mean age of AFO, relative arrangement of the soft tissues, and the stage of development of the involved tooth as the key indicative factors of differentiation between ameloblastic fibroma and fibrodentinoma [8, 9]. Predominantly AFO appears hamartomatous while few others appear to have true neoplastic nature [10].

Patients present with swelling, unerupted tooth, rapid destruction, and cortical plate expansion. This case also presented with marked intraoral and extraoral swelling resulting in the obliteration of molar buccal sulcus and cortical plate expansion. World Health Organization (WHO) also described this well encapsulated tumour to be a benign odontogenic neoplasm admixed in primitive ectomesenchymal tissue exhibiting varying degrees of inductive changes and dental hard tissue formation [11, 12].

Radiographically, this tumour varies from a unilocular or multilocular mixed radio-lucent radiopaque lesion with irregular size and shape to complete radio opacity as in the current case consistent with odontoma formation [13, 14]. Histopathological examination reveals the presence of island and strands of odontogenic epithelium with peripheral columnar cells with palisading nuclei resembling ameloblast and central stellate reticulum-like cells were seen which resembled ameloblastic follicle. The mesenchymal component was fibrous and interspersed with large plump fibroblast resembling dental papilla. The dentin may vary structurally from dentinoid to tubular dentin. Our case histopathological picture was consistent with the above findings [15].

Differential diagnosis of ameloblastic fibroodontoma includes odontoma, ameloblastoma, ameloblastic fibroma, odontoameloblastoma, calcifying epithelial odontogenic tumour, calcifying epithelial odontogenic cyst, and adenomatoid odontogenic tumour. These clinical conditions can be clearly differentiated from ameloblastic fibroodontoma based on radiographs due to the presence of dental hard tissue (enamel and dentin), clinical findings, and prognosis that also supplemented our case diagnosis [16]. Ameloblastic fibroodontoma (AFO) includes a conservative surgical enucleation with the removal of associated tooth to prevent any further recurrences in future as performed in the above case. However, in few cases, the unerupted tooth left behind has shown good prognosis.

## 4. Conclusion

A case of ameloblastic fibroodontoma is rare and unacquainted to a clinician due to underreported number of cases in literature. This article presents an exceptional case report of ameloblastic fibroodontoma (AFO) with emphasis on diagnostic criteria thus emphasizing to all the readers its importance. Treatment of this lesion involves a complete surgical enucleation with removal of associated tooth. Additional cases need to be conveyed into certification so as to have a strong understanding concerning various aspects of this disease.
